# Glucose-dependent effect of insulin receptor isoforms on tamoxifen antitumor activity in estrogen receptor-positive breast cancer cells

**DOI:** 10.3389/fendo.2023.1081831

**Published:** 2023-06-09

**Authors:** Stefania Stella, Michele Massimino, Livia Manzella, Nunziatina Laura Parrinello, Silvia Rita Vitale, Federica Martorana, Paolo Vigneri

**Affiliations:** ^1^ Department of Clinical and Experimental Medicine, University of Catania, Catania, Italy; ^2^ Center of Experimental Oncology and Hematology, Azienda Ospedaliera Universitaria (A.O.U.) Policlinico “G. Rodolico - San Marco”, Catania, Italy; ^3^ Division of Hematology, Azienda Ospedaliera Universitaria (A.O.U.) Policlinico “G. Rodolico-S. Marco”, Catania, Italy; ^4^ University Oncology Department, Humanitas Istituto Clinico Catanese, Catania, Italy

**Keywords:** breast cancer, tamoxifen resistance, insulin receptors, glucose level, gene expression regulation

## Abstract

**Introduction:**

Breast cancer is the most common malignancy in women, and it is linked to several risk factors including genetic alterations, obesity, estrogen signaling, insulin levels, and glucose metabolism deregulation. Insulin and Insulin-like growth factor signaling exert a mitogenic and pro-survival effect. Indeed, epidemiological and pre-clinical studies have shown its involvement in the development, progression, and therapy resistance of several cancer types including breast cancer. Insulin/Insulin-like growth factor signaling is triggered by two insulin receptor isoforms identified as IRA and IRB and by Insulin-like growth factor receptor I. Both classes of receptors show high homology and can initiate the intracellular signaling cascade alone or by hybrids formation. While the role of Insulin-like growth factor receptor I in breast cancer progression and therapy resistance is well established, the effects of insulin receptors in this context are complex and not completely elucidated.

**Methods:**

We used estrogen-dependent insulin-like growth factor receptor I deleted gene (MCF7^IGFIRKO^) breast cancer cell models, lentivirally transduced to over-express empty-vector (MCF7^IGFIRKO/EV^), IRA (MCF7^IGFIRKO/IRA^) or IRB (MCF7^IGFIRKO/IRB^), to investigate the role of insulin receptors on the antiproliferative activity of tamoxifen in presence of low and high glucose concentrations. The tamoxifen-dependent cytotoxic effects on cell proliferation were determined by MTT assay and clonogenic potential measurement. Cell cycle and apoptosis were assessed by FACS, while immunoblot was used for protein analysis. Gene expression profiling was investigated by a PCR array concerning genes involved in apoptotic process by RT-qPCR.

**Results:**

We found that glucose levels played a crucial role in tamoxifen response mediated by IRA and IRB. High glucose increased the IC50 value of tamoxifen for both insulin receptors and IRA-promoted cell cycle progression more than IRB, independently of glucose levels and insulin stimulation. IRB, in turn, showed anti-apoptotic properties, preserving cells’ survival after prolonged tamoxifen exposure, and negatively modulated pro-apoptotic genes when compared to IRA.

**Discussion:**

Our findings suggest that glucose levels modify insulin receptors signaling and that this event can interfere with the tamoxifen therapeutic activity. The investigation of glucose metabolism and insulin receptor expression could have clinical implications in Estrogen Receptor positive breast cancer patients receiving endocrine treatments.

## 1 Introduction

Breast cancer (BC) is the tumor with the highest incidence in women worldwide. According to molecular profiling, BC is a heterogenous disease including different subtypes (1, 2). Among them, Luminal A and B BC, which differ for Ki67 levels and express estrogen and/or progesterone receptor (ER+/PgR+), are the most common, and hormone receptors represent a prognostic and predictive factor (3). Several risk factors associated with BC development have been described comprising molecular alterations (4, 5), obesity (6), glucose metabolism (7), and others (8) involvement, also, the Insulin (Ins)/Insulin-like growth factor (IGF) system (9, 10).

Ins and IGF system comprises a complex intracellular network intricates in cell metabolism, differentiation, and survival and displays a crucial role in several tumors, including BC (11). This system comprises two highly homology types of receptors, insulin-like growth factor receptors (IGFRs) and insulin receptors (IRs). IGFRs are classified into two classes of receptors, insulin-like growth factor receptors I (IGFIR) and II (IGFIIR). IGFIR is catalytically active as tyrosine kinase receptors, while IGFIIR lacks this property (12). IR is a mediator of metabolic and mitogenic effects strongly influencing healthy and cancer cells, especially when overexpressed or in the presence of deregulated glucose metabolism. Two IR isoforms have been described and classified as IRA and IRB according to the absence of exon 11 in isoform A compared to B. IRs and IGFRs can work in concert generating hybrid complex and compensatory intracellular signals (13).

The role of IRs in cancer, including BC, has been reported and related to IRs’ ability to improve tumor progression and chemoresistance, and to modulate the cancer stemness (14–17). In the context of the insulin/IGF system, hyperglycemia and glucose metabolism deregulation have been reported to promote cell proliferation, invasion, migration, and chemotherapy resistance in breast cancer (18, 19).

About 70% of all BC express high levels of ER based on its expression by immunohistochemistry approach. ER-mediated signaling is a carcinogenic driver in ER+ BC patients and its inhibition by endocrine therapies, including tamoxifen (TAM) treatment, improved patients’ survival (20). Published studies also revealed a crosstalk between ER and IR signaling, playing a role in BC progression (21–23).

Using an IGFIR-KO ER+ BC model, selectively expressing IRA or IRB we investigated the role of glucose levels on ER and IRs interplay on drug response using the selective estrogen receptor modulator (SERM) TAM (24, 25).

## 2 Methods

### 2.1 Cell lines and treatment

MCF7 Clustered Regularly Interspaced Short Palindromic Repeats (CRISPR)-Cas9, knock-out (KO)-IGF1R (MCF7^KOIGF1R^) were purchased from Applied Biological Materials (Richmond, BC, Canada) and the selective expression of IRA (MCF7^KOIGF1R-IRA^) were previously generated (14). According to the same procedure previously reported (13), to obtain a cell line selectively expressing IRB isoform (MCF7^KOIGF1R-IRB^), RG215691-hIR-B plasmid (Origene) was used as a template to generate IRB-FLAG by PCR and cloned in pLEX^G418^, while pLEX^G418^EV was used to obtain MCF7^KOIGF1R-EV^.

MCF7^KOIGF1REV^, MCF7^KOIGF1R-IRA^ and MCF7^KOIGF1R-IRB^ cell lines were cultured in DMEM (Sigma) supplemented with 10% fetal bovine serum (FBS) (Euroclone), 2mM glutamine, 50µg/ml streptomycin and 100µg/ml penicillin (all from Sigma).

For all experiments, 30.000/cm^2^ cells for each MCF7 clone were implanted and grown in FBS-free DMEM Low Glucose (LG, 1000mg/L) or DMEM High Glucose (HG, 4500mg/L) containing 0.1% of bovine serum albumin (BSA) (all from Sigma). Then each cell line was grown in DMEM in the presence of LG or HG supplemented with 2,5% of charcoal-stripped FBS, 2mM glutamine, 50µg/ml streptomycin, 100µg/ml penicillin and 1nM of Estradiol (E2) (all from Sigma). Insulin receptor activation was performed using 10nM of Ins (26) while the TAM (Sigma) was used at logarithmic concentration or 10µM as indicated for each experiment for 48 hrs.

### 2.2 IC50 calculation and cell proliferation assay

MTT was used for IC50 calculation and cell proliferation assay. For both experiments cells were exposed to logarithmic concentrations of TAM. MTT assay and IC50 calculation were performed as previously reported (27). IC50 values were calculated setting at 100% the percentage of proliferation after 48 hrs of culture, and logistic non-linear regression was used by Prism Software v8.0. For proliferation assay the percentage of viable cells was obtained, for each condition, setting at 100% the absorbance at 550 nm before TAM exposure.

### 2.3 Apoptosis assay and cell cycle analysis

Apoptosis assay and cell cycle evaluation were performed as previously described (28, 29). Briefly, after 48 h of TAM exposure, cells were collected and washed in phosphate buffered saline (PBS); for apoptosis assay cells were stained using Annexin FITC/7AAD kit according to the manufacturer’s protocol (Beckman Coulter), while for cell cycle analysis cells were fixed in 70% ethanol solution and incubated with ribonuclease A and propidium iodide (both from Sigma). The obtained data were analyzed employing FCS express software v 6.0.

### 2.4 Colony forming unit assay

TAM-treated MCF7^KOIGF1R-EV^, MCF7^KOIGF1R-IRA^ and MCF7^KOIGF1R-IRB^ were detached by trypsin and diluted in identical volumes of medium. An equal volume for each condition was implanted and the cells re-exposed to 10µM TAM. Fresh medium supplemented with insulin was replaced every day for 15-20 days. At this time the cells were stained by crystal violet solution and the colonies were counted employing Image J software.

### 2.5 Western blot and densitometric analysis

Cell pellets were collected, and protein lysate was prepared. Western blot and densitometric analysis were performed as previously reported (30, 31). The primary antibodies used were polyclonal anti-p21^CIP1/waf1^ (Santa Cruz), polyclonal anti-Insulin Receptor (Santa Cruz), polyclonal anti-ERK1/2, anti-pERK1/2^Y204^, anti-pIR^Y1136/1136^ (all from Cell Signaling), monoclonal anti-FLAG and anti-Actin (both from Sigma). Appropriate horseradish peroxidase-conjugated secondary antibodies (Amersham Biosciences) were added and protein expression was then detected using the WesternSure Premium Chemiluminescent Substrate. Immunoblot images were acquired by C-Digit (both from Licor).

### 2.6 Gene Expression Profiling and analysis of Differently Expressed Genes

Cells were collected and used to isolate total RNA employing the RNeasy Plus Mini kit (Qiagen). Extracted total RNA was converted into cDNA using the RT2 First Strand kit (Qiagen) and used to perform RT-qPCR according to the manufactures’ protocol for RT2 Profiler PCR Array Human Apoptosis (Qiagen). DEGs were calculated using a website data analysis software (Qiagen) assigning a threshold ≤2 for genes down-regulated and ≥2 for those up-regulated. Tile plots were generated using Prism Software v 8.0.

### 2.7 Statistical analysis

*Anova* and *t-test* were employed to calculate the p-value by Prism Software v. 8.0 assigning *p<0.05, **p<0.01, and ***p<0.001.

## 3 Results

### 3.1 Cell model validation

Lentivirally transduced cells have been subjected to molecular validation for assessment of the IR isoforms expression and quantification (**Supplementary Figure 1**). To this end, RNA has been isolated from each cell line and reverse transcribed in cDNA which was then used for PCR and RT-qPCR. PCR analysis revealed that MCF7^KOIGF1R-EV^ express predominantly IRB isoform while MCF7^KOIGF1R-IRA^ and MCF7^KOIGF1R-IRB^ over-express preferentially IRA and IRB respectively (**Supplementary Figure 1A**). For IR quantification by RT-qPCR we used the endogenous IR expression of MCF7^KOIGF1R-EV^ as control and 2^-ΔΔCt^ calculation documented that MCF7^KOIGF1R-IRA^ express 7.3 and MCF7^KOIGF1R-IRB^ 6.83 fold higher of IRA or IRB levels respectively, when compared to endogenous IR MCF7^KOIGF1R-EV^ (**Supplementary Figure 1B**).

### 3.2 IRA and IRB reduce the antiproliferative activity of TAM in the presence of HG

**Figure 1:**
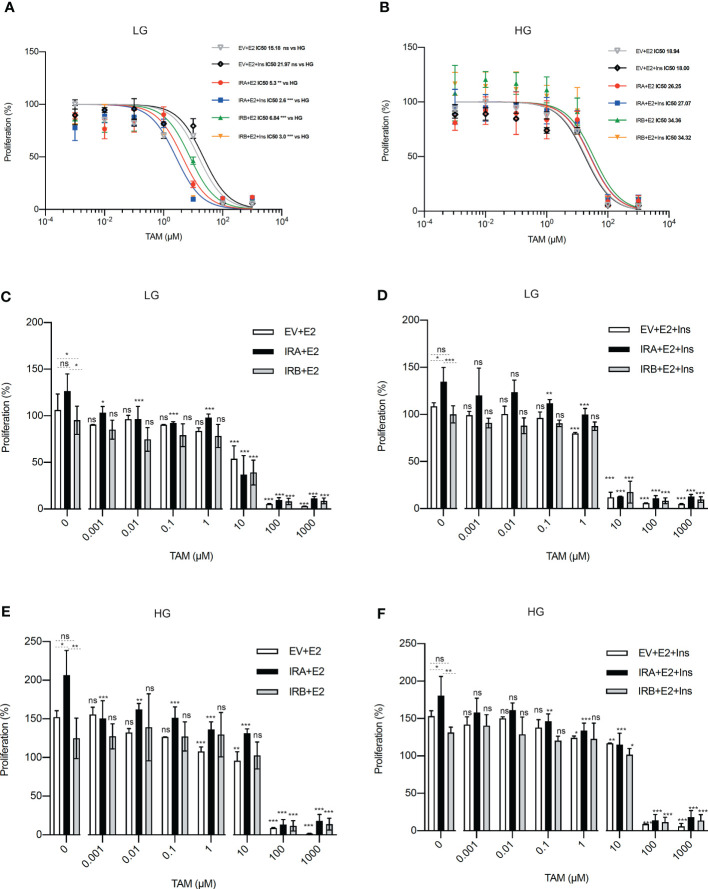
IRA and IRB reduce the TAM activity in the presence of HG. Each cell line was exposed to logarithmic concentrations of TAM and cultured using the indicated conditions of glucose levels, E2 and Ins. **(A, B)** Curves represent the growth rate used to calculate the IC50^TAM^ value by logistic non-linear regression employing Prism Software v8.0. **(C–F)** Histograms showing the percentage of proliferation setting at 100% of the growth rate at the start of the experiment, before to insulin stimulation and TAM exposure, for each condition. For all experiments, the bars indicate the standard deviation derived from two independent experiments performed in duplicate using MTT. Statistical analysis was performed using a t-test to compare the untreated conditions and Anova followed by multiple Tukey comparison tests for the TAM-exposed cells (ns, no significant, *p<0.05, **p<0.01, ***p<0.001).

We wanted to establish the impact of glucose levels on the ability of IRA and IRB to modulate the anti-proliferative activity of TAM. We exposed MCF7^KOIGF1R-EV^, MCF7^KOIGF1R-IRA^ and MCF7^KOIGF1R-IRB^ cells to logarithmic concentrations of TAM, in the presence and absence of insulin, for 48 hrs in a medium containing HG or LG concentration (**Figure 1**). First, we measured the IC50 value of TAM (IC50^TAM^) observing that using LG, both IRA and IRB comparably reduced the IC50^TAM^ when compared with EV expressing cells, an effect that was prominent in presence of insulin stimulation (**Figure 1A**). Interestingly, repeating the same experiment cultivating the cells with HG, we observed that IC50^TAM^ values were higher when compared with EV and LG for either IR isoforms and this effect was independent on insulin exposure (**Figure 1B**). Finally, no differences have been observed between the IC50^TAM^ in EV expressing cells in both LG and HG conditions (**Figures 1A, B**). Next, applying the same experimental conditions reported above, we measured the cell proliferation after TAM exposure setting at 100% of the proliferation rate observed at the start of the experiment before exposing the cells to TAM alone or combined with insulin. Regardless of insulin stimulation and glucose levels, IRA induced cell growth more consistently than EV and IRB (**Figures 1C–F**, left columns). Moreover, although IRA showed this same effect after treatment, it increased the sensitivity to TAM exposure in all experimental conditions. In fact, IRA displayed a statistically significant proliferation reduction following the augmentation of TAM concentration, an event that was not overserved for EV and IRB, which displayed cytostatic effect (**Figures 1C–F**, middle columns). Using higher concentrations of TAM, we found that the critical dose was 10µM, which generated different results according to both the glucose levels and insulin stimulation. Using LG, insulin increased the cytotoxicity of 10µM TAM (**Figures 1C, D**, right columns), which, in turn, was less effective when the cells were cultured in the presence of HG (**Figures 1E, F**, right columns). No different effect was detected using TAM 100µM and 1000µM. Furthermore, to investigate if 10µM TAM did not exert ER independent activity (32), we performed IC50^TAM^ calculation on MDA-MB-468 triple-negative breast cancer cells, finding a higher IC50^TAM^ value when compared to EV, IRA, and IRB expressing cells (**Supplementary Figure 2**).

Overall, these data support the role of both IRA and IRB on the modulation of TAM cytotoxicity, critically regulated by glucose levels and insulin stimulation.

### 3.3 IRB displays anti-apoptotic activity and promotes cell cycle arrest according to the glucose levels and insulin stimulation after TAM exposure

**Figure 2:**
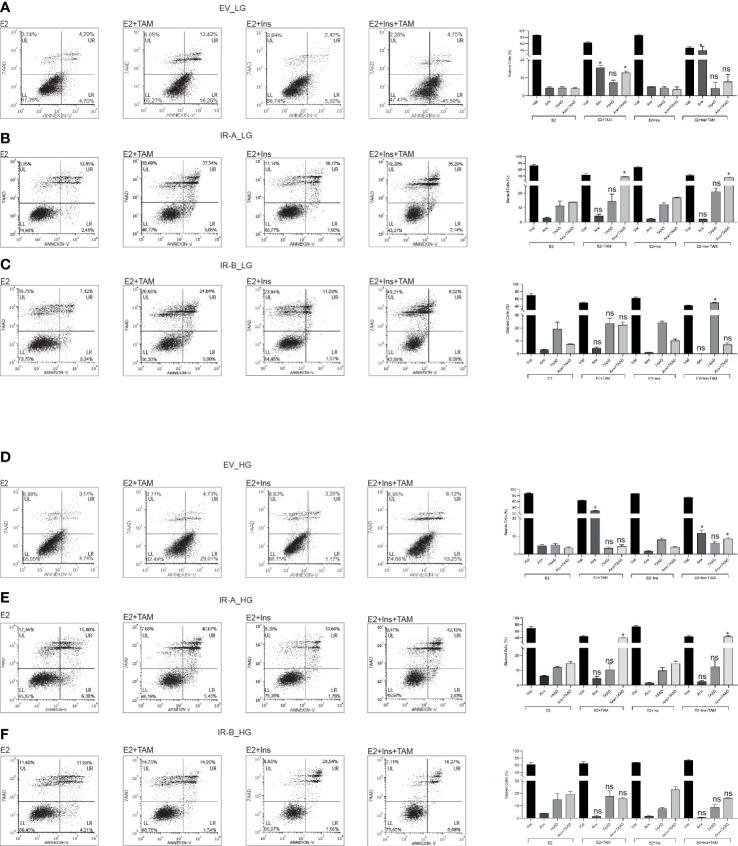
HG promotes the anti-apoptotic activity of IRB after TAM treatment. MCF7^KOIGFIR-EV^, MCF7^KOIGFIR-IRA^ and MCF7^KOIGFIR-IRB^ cell lines were exposed or not to 10µM TAM and cultured as indicated. Then, cells were collected and stained by Annexin-V (Anx) and 7AAD to evaluate the apoptosis rate in LG **(A–C)** or HG **(D–F)** levels. Histograms report the percentage of vital (LL quadrant), Anx (LR quadrant), 7AAD (UL quadrant), or Anx+7AAD (UR quadrant) stained cells, and bars indicate the standard deviation obtained from two independent experiments performed. Anova followed by multiple Tukey comparison tests was performed on a percentage of cells stimulated with insulins compared to the same experimental condition without insulin (ns, no significant, *p<0.05) (LL: lower left, LR: lower right, UL: upper left, UR: upper right).

**Figure 3:**
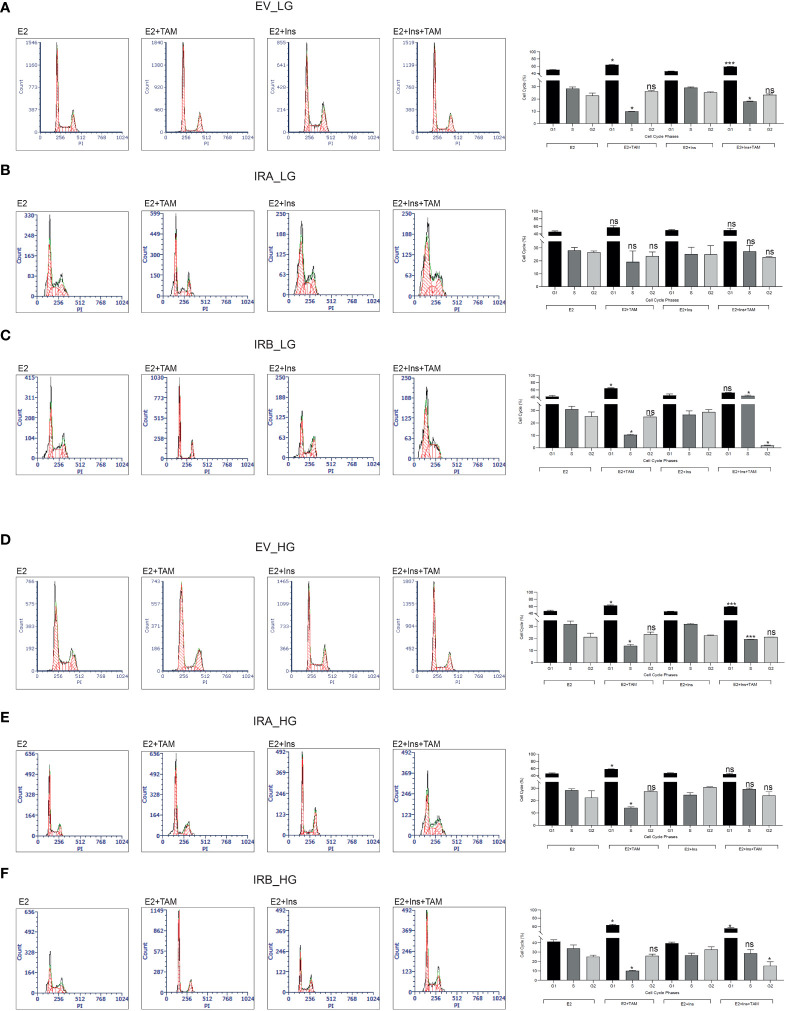
IRB promotes cell cycle arrest after TAM exposure independent of glucose levels. The indicated untreated and 10µM TAM-treated BC cells were collected and stained using propidium iodide. Cells were cultured as reported in the presence of LG **(A–C)** or HG **(C–E)**. Histograms indicate the percentage of cell cycle distribution for each indicated experimental condition. Bars indicate the standard deviation obtained from two independent. Anova followed by multiple Tukey comparison tests was performed on a percentage of cells stimulated with insulins compared to the same experimental condition without insulin (ns, no significant, *p<0.05, ***p<0.001).

Next, we wanted to determine if the observed effects of IRA and IRB on cell proliferation after TAM exposure were dependent on apoptosis and/or cell cycle regulation (**Figures 2**, **3**). We exposed MCF7^KOIGFIR-EV^, MCF7^KOIGFIR-IRA^ and MCF7^KOIGFIR-IRB^ cell clones to TAM 10µM for 48 hrs in the presence or absence of insulin. Using LG levels, both EV- and IRA-expressing cells were susceptible to the TAM cytotoxicity, the effect that was independent of insulin stimulation (**Figures 2A, B**), while IRB expression arrested the TAM-induced apoptosis, but only in the absence of insulin. However, we observed an increased necrosis rate in IRB-expressing cells, that was statistically significant after insulin stimulation (**Figure 2C**). In the presence of HG and regardless of insulin stimulation, EV and IRA failed again to protect the cells from TAM cytotoxicity, while IRB promoted anti-apoptotic activity in all experimental conditions (**Figures 2D–F**).

These results suggest that IRA was less effective than IRB to reduce TAM toxicity and that these effects were not dependent on glucose levels or insulin stimulation. On the contrary, IRB activity was dependent on both insulin and glucose concentration.

We then investigated cell cycle progression for each cell line after 10µM TAM exposure for 48 hrs. We found that TAM cytotoxicity was strongly influenced by glucose levels and insulin exposure (**Figure 3**). By culturing the cells with LG we detected a significant reduction of the S phase in EV-expressing cells after TAM exposure both in the absence and presence of insulin stimulation, an effect that was documented by increasing of G1 phase (**Figure 3A**). We did not observe a statistically significant modification in cell cycle distribution for IRA expressing cells in all experimental conditions (**Figure 3B**), suggesting that IRA promoted cell cycle progression even in presence of TAM. Interestingly, IRB showed a dual block mode of cell cycle arrest dependent on its activation by insulin. Without insulin, IRB increased the G1 phase followed by S phase reduction, an effect that was reverted by insulin producing a strong increase of the S phase, and both effects were statistically significant (**Figure 3C**). Compared to LG, HG levels did not modify the cell cycle modulation of EV, while it altered that mediated by IRA and IRB. In the absence of insulin both IRA and IRB, induced a statistically significant cell cycle block increasing G1 and reducing S phases, an event that was reverted by insulin for IRA but not for IRB. In fact, IRB preserved this effect after insulin stimulation but only for the G1 phase. Both effects were statistically significant when compared with the condition in the absence of insulin exposure (**Figures 3C–E**).

Interestingly, these results implicate a crucial role of association between glucose levels and insulin stimulation for both insulin receptors that differently regulated apoptosis and cell cycle distribution.

### 3.4 IRB increases the BC cells fraction able to retain clonogenicity properties after TAM exposure

**Figure 4:**
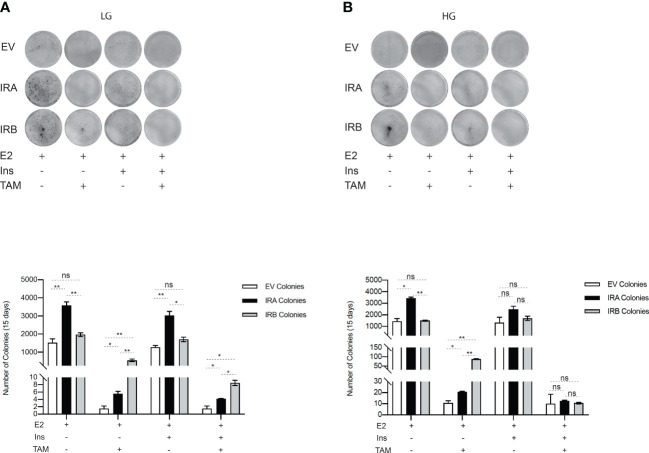
IRB, differently, preserves the clonogenicity of TAM-survived cells according to LG or HG. MCF7^KOIGFIR-EV^, MCF7^KOIGFIR-IRA^ and MCF7^KOIGFIR-IRB^ untreated and survived to 10µM TAM were exposed again to 10µM TAM and implanted on 6-well to perform long-term culture by clonogenic assay. Cells were maintained in LG **(A)** or HG **(B)**. The image reports the colonies stained by cristal violet after 15-20 days of culture. Histograms report the number of colonies obtained using Image J software. Bars indicate the standard deviation obtained from two independent experiments. t-test comparison was used to compare a number of different colonies obtained from IRA and IRB for each indicated experimental condition (ns, no significant, *p<0.05, **p<0.01).

We next wanted to evaluate the ability of IRA and IRB to preserve the surviving cells after the first TAM exposure, comparing them with EV condition. We performed a long-term culture by CFU assay for all MCF7 clones measuring their clonogenicity properties by exposing each cell line to 10µM TAM for 48 hrs and when implanted at the start of the long-term culture (**Figure 4**). We found that, in LG untreated condition supplement or not by insulin, IRA was more clonogenic than EV and IRB while after TAM exposure the effect was dependent on insulin stimulation. Indeed, IRA and EV expressing cells stimulated or not by insulin were less efficacy, than IRB, to preserve the vital cells that survived at the first TAM exposure, while IRB, showed a statistically significant ability to save the BC cells fraction able to colonies generation, especially in absence of insulin (**Figure 4A**). Different results were observed when we repeated the same experiment using HG levels. In the absence of insulin, IRA produced a higher number of colonies than IRB, an effect that was restored by insulin stimulation, where the number of colonies generated by two receptors was comparable and not statistically significant. Finally, IRB preserved the clonogenicity of MCF7^KOIGFIR-IRB^ only when we exposed it to 10µM TAM in the absence of insulin. EV-expressing cells showed reduced clonogenicity than IRA and IRB in all experimental conditions independent of insulin stimulation and TAM exposure (**Figure 4B**).

Altogether our results support that IRB promotes longer cell survival in response to TAM according to glucose levels and its occupied/unoccupied state.

### 3.5 IRB modulates the ERK1/2 phosphorylation and p21CIP1/WAF1 expression in response to TAM treatment and glucose levels

**Figure 5:**
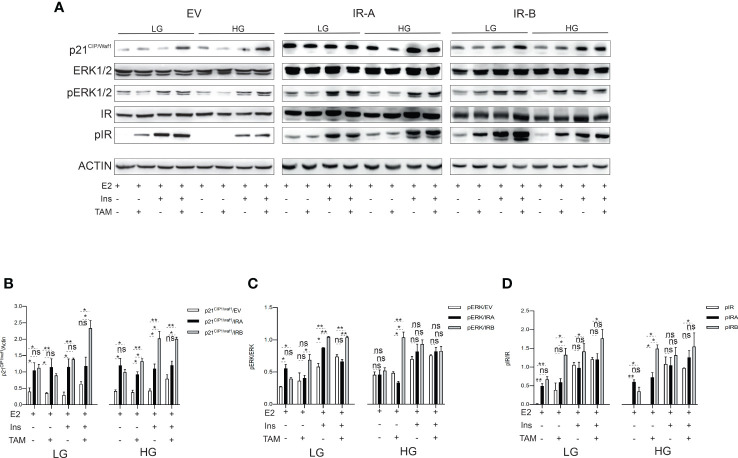
IRB induces p21^CIP1/waf1^ and ERK1/2 phosphorylation according to glucose levels and insulin stimulation after TAM exposure. MCF7^KOIGFIR-IRA^, MCF7^KOIGFIR-IRA^ and MCF7^KOIGFIR-IRB^ were cultured as indicated. Untreated or exposed to 10μM TAM were collected after 48 hrs and protein lysates have been prepared. SDS-page was used for protein separation and the nitrocellulose membrane was stained using the primary antibodies directed against the indicated proteins **(A)**. Immunoblot image was used to measure the different protein expressions by densitometric analysis employing Image J software, for p21^CIP1/waf1^ **(B)**, pERK1/2 **(C)**, and pIR **(D)**. The densitometric unit value was obtained by normalizing the phospho- and total- protein by actin, then the ratio phospho-/total- protein was used for histogram generation. For endogenous IR expression (EV) measuring an anti-insulin receptor antibody was used, while anti-FLAG antibody was employed to detect the expression levels of recombinant IR (IRA, IRB). Bars indicate the standard deviation obtained from two independent experiments. t-test comparison was used to compare different densitometric levels for each indicated experimental condition (ns, no significant, *p<0.05, **p<0.01).

Given previous findings, we wanted to establish whether a relationship exists between IRs-mediated proliferation, cell cycle, and apoptosis modulation and p21^CIP1/WAF1^ or ERK1/2 proteins, two well-established mediators of these intracellular events (33, 34). We exposed each breast cancer cell to 10µM TAM in both LG and HG growth medium supplemented or not by insulin for 48 hrs. Then, an immunoblot was performed to analyze the ERK1/2 phosphorylation levels and p21^CIP1/WAF1^ expression. Moreover, as it was demonstrated that IRs activity is regulated by its ligand-occupied and unoccupied state (26), we also evaluated the phosphorylation levels of both insulin receptors (**Figure 5**).

Densitometric analysis (**Figure 5A**), revealed that the p21^CIP1/waf1^expression and ERK1/2 phosphorylation have been differently modulated by TAM exposure in EV, IRA and IRB-expressing cells.

In the presence of LG, IRA and IRB increased the basal expression p21^CIP1/waf1^ when compared to EV. However, IRB, strongly, induced p21^CIP1/waf1^ once the cells have been exposed to TAM and insulin combination more than IRA. Interestingly, this phenomenon was preserved in the presence of HG, which also, promoted this effect after TAM exposure in both the presence and absence of insulin stimulation, while no difference in p21^CIP1/waf1^ expression has been observed between the two receptor isoforms in untreated conditions. Finally, EV showed a weak induction of p21^CIP1/waf1^ and only in the presence of TAM (**Figure 5B**).

When we analyzed the ERK1/2 phosphorylation levels we found that the glucose levels played a crucial role to determine the IRA- and IRB-mediated effects. When the glucose levels were low, IRB was able to promote the ERK1/2 phosphorylation more prominently than IRA, both in the presence of insulin alone and combined with TAM. Surprisingly, in the absence of insulin and after TAM treatment, IRB determined the same effect especially when the cells were cultured in HG conditions. Finally, the ERK1/2 phosphorylation levels were comparable between two insulin receptors after insulin exposure alone or combined with TAM in the HG medium. No difference in ERK1/2 phosphorylated state was observed in both LG and HG untreated conditions between the two receptor isoforms. In EV-expressing cells, ERK1/2 phosphorylation levels were induced only in the presence of insulin and have been less prominent than IRA and IRB (**Figure 5C**).

As IR-mediated intracellular signal is dependent on the occupied and unoccupied state of IRs (35, 36), we evaluated their phosphorylation levels following or not insulin exposure. Unexpectedly, we found that in the absence of insulin and after TAM exposure, IRB was activated. This event was independent of glucose levels and statistically significant when compared to IRA. The phosphorylation levels of both IRA and IRB were induced by insulin stimulation in both LG and HG conditions regardless of TAM exposure, and although IRB showed higher phosphorylation levels than IRA, these were not statistically significant. In untreated conditions, no difference between the two insulin receptors has been observed. In the EV condition, which represents the endogenous state of the insulin receptor, the insulin-mediated IR activation was less prominent than IRB but comparable with IRA, while weak or no phosphorylation was observed in the absence of insulin (**Figure 5D**).

Overall, these findings support a different association of p21^CIP1/waf1^ and ERK1/2 involvement with occupied and unoccupied states of both IRA and IRB under TAM exposure.

### 3.6 Glucose levels differently modify the GEP mediated by activated IRA and IRB regulating an apoptotic network associated with TAM exposure

**Figure 6:**
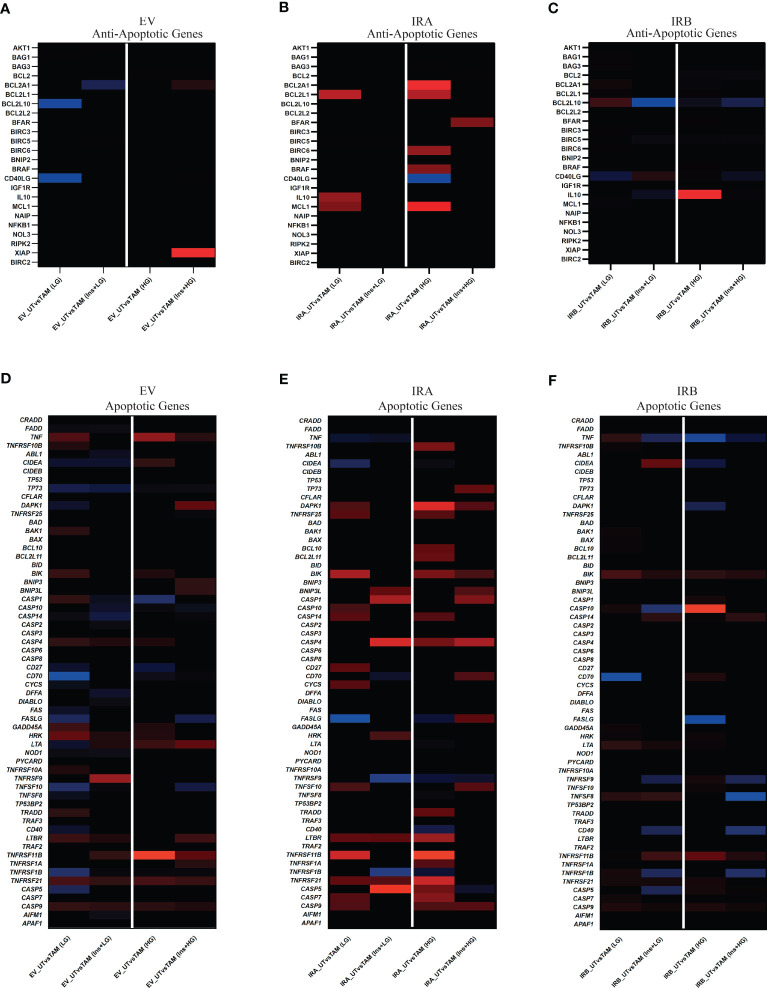
IRA and IRB insulin stimulation induce a GEP profiling revealing a direct involvement of TNF, BCL2, and CASP gene family. MCF7^KOIGF1R-EV^, MCF7^KOIGF1R-IRA^ and MCF7^KOIGF1R-IRB^ were cultured in LG or HG glucose in the presence of E2 and exposed or not with TAM and/or Ins as indicated. Total RNA was isolated and used for cDNA generation. RT-qPCR was used to quantify the anti- **(A–C)** and pro-apoptotic **(D–F)** genes using untreated conditions as control for each cell line. Tile plots show the lower expressed (threshold ≤2, blue) and over-expressed (threshold ≥2, red) of DEG for each subset of analyzed genes.

To analyze the HG and LG effects on GEP following insulin stimulation we exposed or not MCF7^KOIGF1R-EV^, MCF7^KOIGF1R-IRA^ and MCF7^KOIGF1R-IRB^ cells to 10µM TAM for 48 hrs. Then, total RNA was isolated, and RT-qPCR was performed to evaluate the expression of a subset of anti-apoptotic and apoptotic genes (**Supplementary Table 1**) in TAM-treated cells using the untreated condition as the control for each cell line. Fold change of ≥2 or ≤2 was used as a cutoff to classify the up- and down- regulated genes (**Figure 6**).

Independent of glucose concentrations we observed that TAM exposure followed or not by insulin stimulation showed a weak effect on the modulation of anti-apoptotic genes subset in both EV and IRB-expressing cells. Otherwise, insulin exposure reverted the TAM-mediated effect on anti-apoptotic genes in cells over-expressing IRA isoform (**Figures 6A–C**). When we analyzed the modulation of apoptotic genes in all cell lines, the effects have been dependent on glucose levels and insulin stimulation. In MCF7^KOIGFIR-EV^ cells, we observed a weak over-expression of apoptotic genes in all experimental conditions that were predominant when we cultivated the cells in HG (**Figure 6D**). Interestingly, the over-expression of IRA or IRB generated a strong modulation of apoptotic genes. In MCF7^KOIGFIR-IRA^ cells, the over-expression of IRA increased apoptotic genes expression levels, an effect that was weakly reduced once the cells have been exposed to insulin stimulation in both LG and HG growth conditions (**Figure 6E**). Otherwise, the over-expression of IRB induced a very weak expression of apoptotic genes, an effect that was reverted after insulin stimulation where a strong down-regulation of these genes have been observed (**Figure 6F**; **Supplementary Figure 3**).

Overall, these findings suggest that the signal transduction pathways following IRA and IRB activation is critically dependent on glucose levels, thus mediating a different biological response to TAM exposure with a predominant apoptotic effect by IRA and anti-apoptotic activity by IRB.

## 4 Discussion

Extensive pre-clinical and clinical results support the crucial role of the insulin/IGF system on breast cancer development, chemoresistance, and progression (9). However, IGFIR inhibition did not prove effective in the treatment of breast cancer so far. This lack of activity potentially relies on the crosstalk between IGFIR, and IR reported in several tumor types, including BC (15, 37, 38). Indeed, BC cells can overcome the IGFIR block, processing the intracellular signaling as a result of a compensatory mechanism (15). In addition, IR signaling is strongly associated with glucose levels (39) and estrogen receptor-mediated pathways in BC (23). Furthermore, previously published data reported the effect of hyperglycemia on chemoresistance (39) and TAM exposure (40) but none of them reported the role of IR isoforms in this field.

In this study, we investigated the role of glucose levels on IRA and IRB activity on ER+ BC cells exposed to TAM. To avoid the compensatory mechanisms mediated by the IGFIR-IRs system, we used an IGFIR-KO BC model preferentially expressing IRA or IRB that we identified as MCF7^KOIGF1R-IRA^ and MCF7^KOIGF1R-IRB^ BC cells comparing the observed effects with cells expressing endogenous levels of IR named MCF7^KOIGF1R-EV^.

We observed that HG concentrations increased the IC50^TAM^ and reduced the TAM-dependent proliferation arrest. Although IRA was more mitogenic than IRB (26, 41), it unexpectedly showed reduced resistance to TAM cytotoxicity when compared to IRB, which was able to promote a cytostatic effect. However, using a high dose of TAM both receptors showed comparable results which were dependent on glucose levels and insulin stimulation. Surprisingly, we detected that insulin promoted TAM activity in LG conditions while HG levels predominantly induced resistance to TAM especially in IRB-expressing cells.

Previously published data reported that the modulation of apoptosis by insulin receptors is associated with their ligand-occupied or unoccupied state (26, 42). When we analyzed the apoptotic response to TAM, we observed that it was deeply modified by glucose concentrations regardless of the occupied or unoccupied state of both insulin receptors. Both in the presence of LG and HG, IRA was less protective than IRB. In turn, IRB completely blocked the cytotoxic effect of TAM, independent of its insulin stimulation especially when the glucose levels were high. Although these data seem in contrast with what was previously reported (26, 42), they show that glucose levels may modify insulin receptors activity, playing a critical role in TAM response in BC.

Subsequently, by cell cycle analysis we observed that, unlike IRA, IRB induced cell cycle block strongly suggesting that the observed anti-apoptotic activity was dependent on its ability to save the cells inducing their proliferation arrest. These observations are supported by data obtained with IRA, which promoting cell cycle progression was not able to preserve the cell survival from TAM cytotoxicity. Indeed, according to pre-existing evidence, cell cycle block is a survival mechanism used by tumor cells to escape from apoptosis induced by different therapeutic agents (43, 44).

The clonogenic assay is an *in-vitro* method used to evaluate the long-term culture cell survival based on the ability of a single cell to generate a colony (45). We used this method to measure the ability of IRA and IRB to preserve the BC cell fraction, survived the first TAM exposure, to retain the clonality expansion properties when exposed again to TAM. Regardless of its activation by insulin treatment, IRA was more clonogenic than IRB but less effective to preserve the surviving cells after the first TAM exposure. These findings are consistent with those obtained by cell cycle experiments, suggesting that by inducing cell cycle arrest, IRB promoted the long-term survival of BC cells. These findings are supported by IRB-mediated p21^CIP1/waf1^ induction, as previously reported (46). Moreover, ER inhibition by TAM promotes CDK4/6 block, which can be overcome by p21^CIP1/waf1^ resulting in cell survival (47, 48).

ERK1/2 activation is generally associated with both cell survival and apoptosis. This event is based on published observations reporting that the balance between the intensity and duration of both apoptotic and survival signals involving ERK1/2 determines the cell fate (49). We found that both insulin receptors induced ERK1/2 activation. However, only in IRA-expressing cells did the proliferative effect occurr, suggesting that in IRB-expressing cells, the p21^CIP1/waf1^ induction was the predominant intracellular signal that caused cell cycle arrest (50). As expected, IR phosphorylation levels were induced by insulin (42). However, IRB showed activation even if in the absence of insulin but after TAM exposure. Phosphorylated IRB induced ERK1/2 activation but not p21^CIP1/waf1^ induction, suggesting an IRB kinase-independent mechanism.

**Figure 7:**
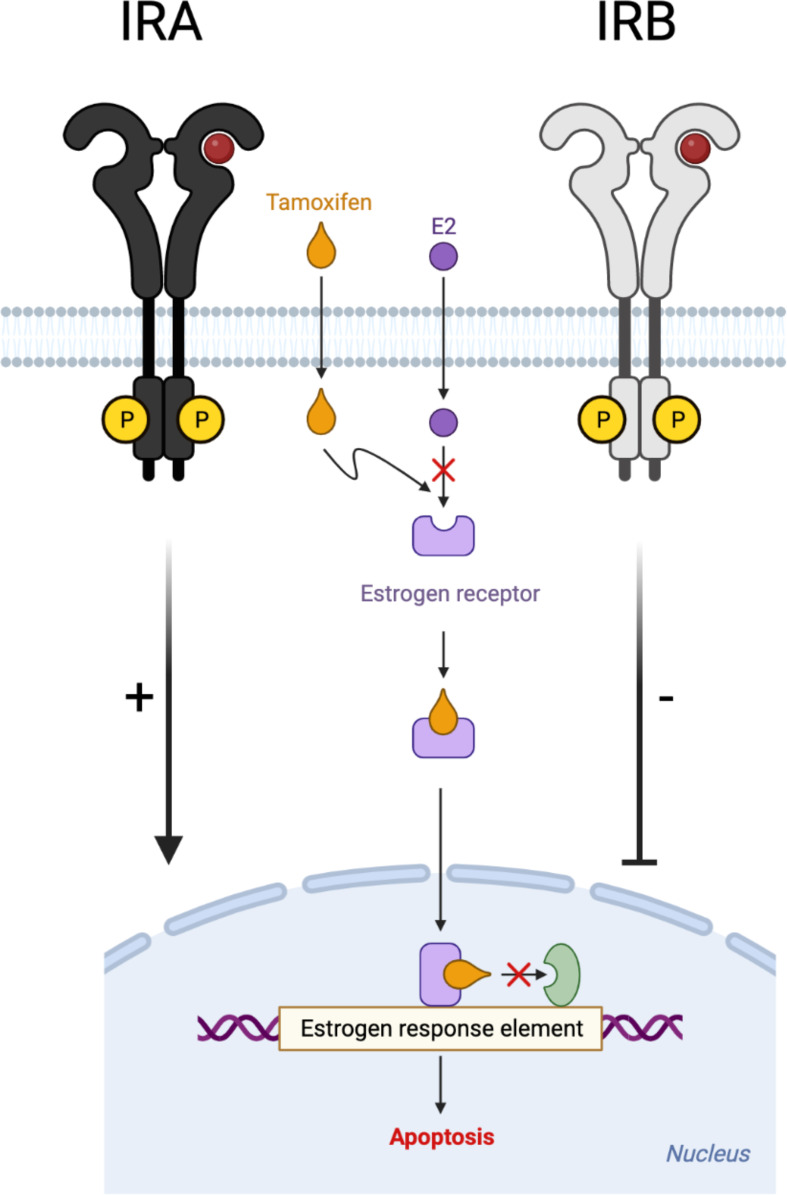
Schematic representation of IRA and IRB role on TAM-induced cytotoxic effect. Different modulation of insulin receptor isoforms **(A, B)** on TAM-induced cytotoxicity in estrogen-dependent breast cancer cells.

Extensive published data report that the decision of cell fate is strongly dependent on the balance between pro- and anti-apoptotic stimuli (51–53). GEP analysis revealed that anti-apoptotic genes have not been significantly modified for except IRA expressing cells where they have been over-expressed by TAM alone, while after insulin stimulation this effect was lost. Different effects have been observed for apoptotic genes, especially for IRA- and IRB-expressing cells. Indeed, while IRA showed a strong increase in these genes, IRB induced a down-regulation which was prominent after insulin stimulation. Our results suggest that in the presence of IRB, the apoptotic signal was down-regulated while it was up-regulated by IRA (**Figure 7**). Interestingly, both events were strongly influenced by glucose levels and insulin stimulation, suggesting a critical role of glucose metabolism in both tumor cells (54).

In conclusion, we demonstrate the crucial role of the occupied and unoccupied state of both insulin receptors isoforms in response to TAM in the ER+ BC cell model. Based on these cell culture results, we suggest that the deregulation of glucose metabolism associated with IRA and IRB activation could be investigated as a possible predictive biomarker for treatment with TAM in ER+ BC patients.

## Data availability statement

The original contributions presented in the study are included in the article/**Supplementary Material**. Further inquiries can be directed to the corresponding author.

## Author contributions

Conceptualization: MM and SS; Formal Analysis: MM, SS and NLP; Investigation: SS, MM, LM, FM, and SV; Data Curation: SS, MM and LM; Writing - Original Draft Preparation: MM and SS; Writing - Review and Editing: SS, MM, LM, SV, FM, NLP and PV; Supervision: PV. All authors contributed to the article and approved the submitted version.
