# Supplementary material for: Glucose-dependent effect of insulin receptor isoforms on tamoxifen antitumor activity in estrogen receptor-positive breast cancer cells
Source: Front Endocrinol (Lausanne). 2023 Jun 9;14:1081831. doi: 10.3389/fendo.2023.1081831 (PMC10289407; doi:10.3389/fendo.2023.1081831)
Supplement: Supplementary file 1 [file Image_1.pdf]

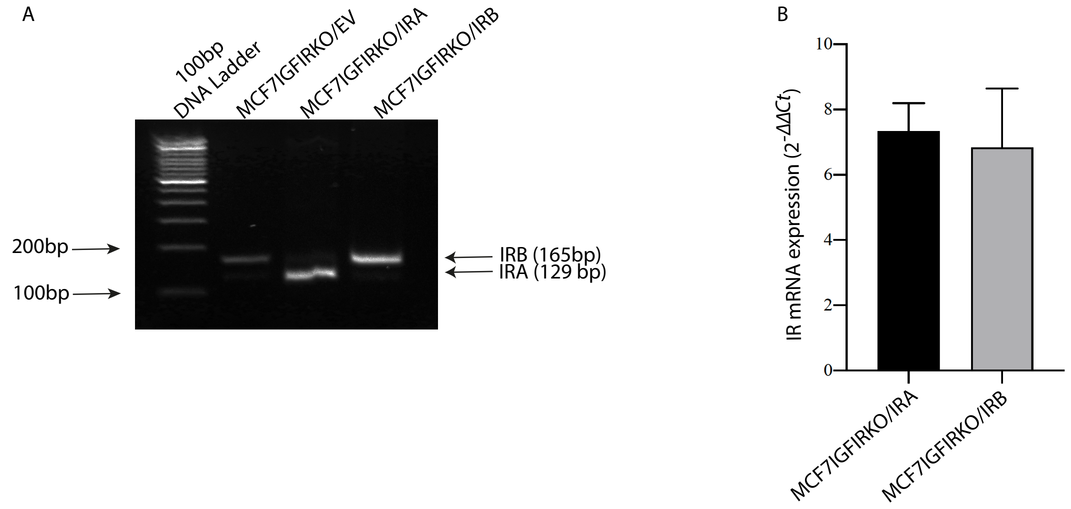

Supplementary Figure 1: MCF7 cells expressing EV, IRA or IRB were used for RNA isolation employing RNeasy Plus Mini kit (Qiagen). RNA was then reverse transcribed using M-MLV enzyme and random examers. The obtained cDNA was then amplified by PCR using Taq Polimerase (A) or quantified by RT-qPCR (B) using PowerUp SYBER Green Mix (both from Thermo Fisher Scientific), both employing specific primers able to discriminate IRA and IRB isoforms (Iso human IR_Fw CCAAAGACAGACTCTCAGAT, Iso humanIR_Rv AACATCGCCAAGGGACCTGC). IR quantification was calculated using MCF7IGFIRKO/EV as control.
