# Supplementary material for: Glucose-dependent effect of insulin receptor isoforms on tamoxifen antitumor activity in estrogen receptor-positive breast cancer cells
Source: Front Endocrinol (Lausanne). 2023 Jun 9;14:1081831. doi: 10.3389/fendo.2023.1081831 (PMC10289407; doi:10.3389/fendo.2023.1081831)
Supplement: Supplementary file 2 [file Image_2.pdf]

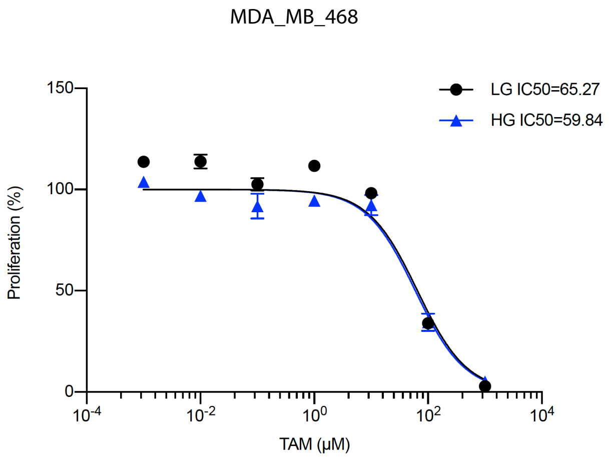


**Supplementary Figure 2:** MDA_MB_468 cells cultivated in presence of low (LG) or high (HG) glucose levels were exposed to logarithmic concentrations of tamoxifen (TAM) for IC50TAM calculation. Curve represent the growth rate used to calculate the IC50TAM value by logistic non-linear regression employing Prism Software v8.0.
