# Supplementary material for: Glucose-dependent effect of insulin receptor isoforms on tamoxifen antitumor activity in estrogen receptor-positive breast cancer cells
Source: Front Endocrinol (Lausanne). 2023 Jun 9;14:1081831. doi: 10.3389/fendo.2023.1081831 (PMC10289407; doi:10.3389/fendo.2023.1081831)
Supplement: Supplementary file 3 [file Image_3.pdf]

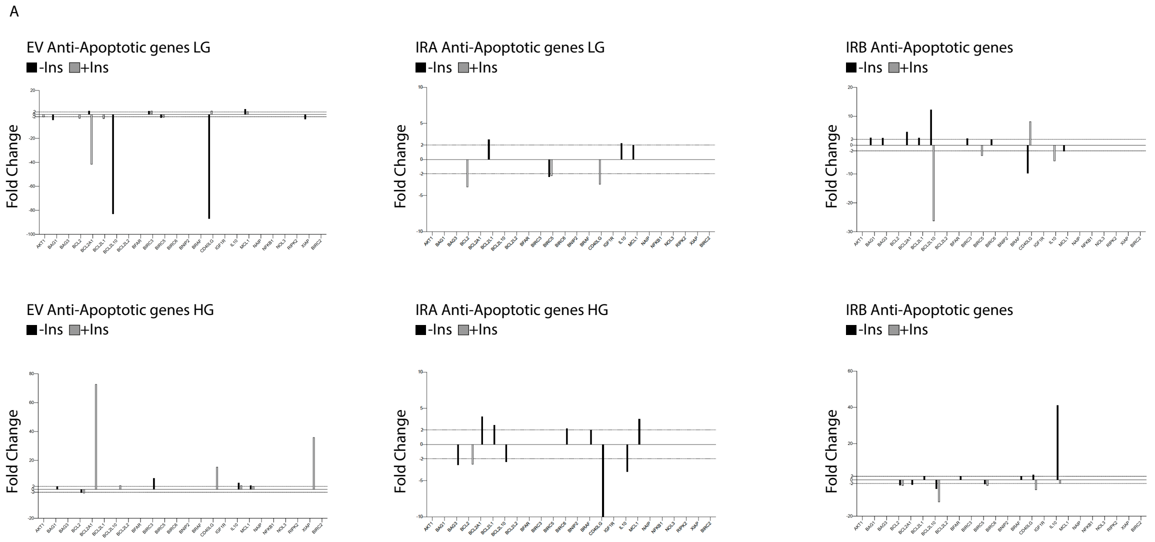

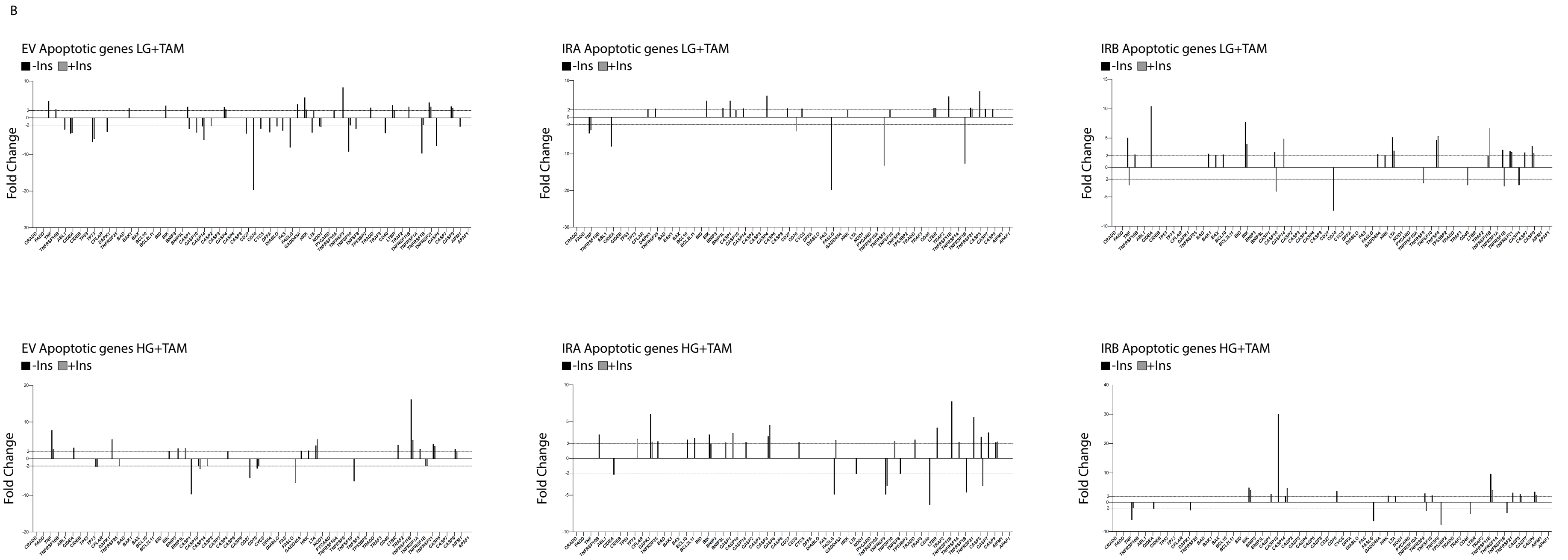

Supplementary Figure 3: Graphs showing the up- and down-regulated anti-apoptotic (A) or apoptotic (B) genes of TAM exposed cells cultivated in LG or HG levels and stimulated or not by 10nM of insulin. The GEP was obtained assigning a threshold <2 for genes down-regulated and >2 for those up-regulated using EV expressing cells as control.

≤!
